# Ambient seafloor noise excited by earthquakes in the Nankai subduction zone

**DOI:** 10.1038/ncomms7132

**Published:** 2015-01-30

**Authors:** Takashi Tonegawa, Yoshio Fukao, Tsutomu Takahashi, Koichiro Obana, Shuichi Kodaira, Yoshiyuki Kaneda

**Affiliations:** 1 Research and Development Center for Earthquake and Tsunami, Japan Agency for Marine-Earth Science and Technology, 3173-25, Showa-machi, Kanazawa-ku, Yokohama 236-0001, Japan; 2 Disaster Mitigation Research Center, Nagoya University, Furo-cho, Chikusa-ku, Nagoya 464-8601, Japan

## Abstract

Excitations of seismic background noises are mostly related to fluid disturbances in the atmosphere, ocean and the solid Earth. Earthquakes have not been considered as a stationary excitation source because they occur intermittently. Here we report that acoustic-coupled Rayleigh waves (at 0.7–2.0 Hz) travelling in the ocean and marine sediments, retrieved by correlating ambient noise on a hydrophone array deployed through a shallow to deep seafloor (100–4,800 m) across the Nankai Trough, Japan, are incessantly excited by nearby small earthquakes. The observed cross-correlation functions and 2D numerical simulations for wave propagation through a laterally heterogeneous ocean–crust system show that, in a subduction zone, energetic wave sources are located primarily under the seafloor in directions consistent with nearby seismicity, and secondarily in the ocean. Short-period background noise in the ocean–crust system in the Nankai subduction zone is mainly attributed to ocean-acoustic Rayleigh waves of earthquake origin.

## Abstract (web summary)

Excitations of seismic background noise are mostly related to fluid disturbances, and earthquakes have not been considered as a source due to their intermittent nature. Here, the authors use a seabed hydrophone array and show that background noise offshore Japan is mainly due to small earthquakes.

## Graphical abstract

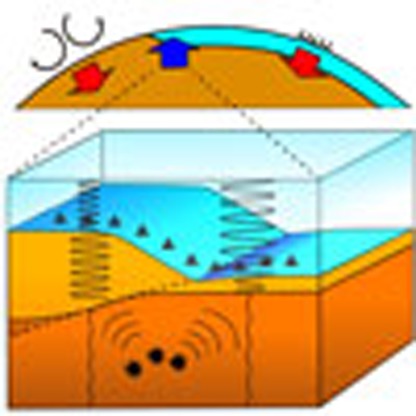

Background noise and its excitation source have been explored over the atmosphere, ocean and solid Earth^1^,^2^,^3^,^4^. Earth’s free oscillations^5^,^6^,^7^ and microseisms^8^,^9^ are excited by random, stationary motions in fluid media, such as atmospheric turbulence^6^ and ocean swells^8^, below 0.2 Hz (Fig. 1a). At higher frequencies, the local microseisms can be generated intermittently in the ocean by strong winds associated with storms^10^,^11^. However, little is known about the existence of incessant excitation at this frequency in calm regions and during quiet seasons.

Acoustic-coupled Rayleigh (ACR) waves^12^ generated by earthquakes have been observed on the seafloor^13^,^14^,^15^. Generation of ACR waves requires the presence of low-velocity marine sediments within which several wavelengths of short-period Rayleigh modes can be contained (<5 Hz^15^). Since the energy of such waves is partitioned into ocean and marine sediments with mutually strong coupling, the wave is not easily attenuated, resulting in long-distant propagation, for example, ~10^4^ km (refs 14, 15), in instances of large earthquakes. Non-attenuation of the waves implies that, if the excitation sources responsible for the ACR waves are continuously and randomly activated, such waves can be seen as background noise on the seafloor.

Ambient noise-correlation techniques may provide key information about these noise sources. Quiet portions of time series (that is, ambient noise) have been recently reported to contain various signals, such as surface and body waves^16^,^17^,^18^. The noise-correlation techniques are capable of retrieving these signals propagating between two positions (stations) in a background wavefield. If the ACR wave can be extracted by correlation techniques, the existence of the source persistently exciting the wave is implicitly indicated.

From September to December 2011, an array of 153 stations with a station spacing of 5 km was deployed around the Nankai Trough, Japan^19^ (Fig. 1b). Each station consists of a hydrophone and a three-component, short-period (4.5 Hz) seismometer. The water depth varied in the range 100–4,800 m in the region where the array was installed. The thickness of the sediment layer approaches to 5–6 km owing to an accretionary prism that thickens in the north of the Nankai Trough^20^. *V*p*/V*s of the sediments is estimated to be approximately 2 to the north of the trough, and 4 to the south of the trough^21^. *V*p and *V*s represent velocities of P and S waves, respectively. Since the *V*p variation in marine sediment is considered to be smaller compared with the *V*s variation^21^,^22^, the characteristics of the ACR wave are sensitive to water depth, *V*s and the thickness of the sediments.

In this study, using the continuous hydrophone record, we calculate cross-correlation functions (CCFs) at a frequency range of 0.7–2.0 Hz, and stack them over the observation period. From the result, we demonstrate that obtained CCFs represent the retrieval of persistently excited ACR waves, and the direction in which strong ACR waves propagate coincides with seismicity around the Nankai subduction zone. Moreover, two-dimensional (2D) numerical simulations indicate that excitation sources responsible for incessant ACR waves are primarily located under the seafloor.

## Results

### Detection of persistently excited ACR waves in the CCFs

Figure 2 and Supplementary Fig. 1a–c show CCFs using two adjacent stations along Lines A–E (Methods). The obtained signals from the northern and southern parts of each line were different, in spite of the same separation distance of 5 km. For instance, in the northern part of Line A (Fig. 2a), a strong signal with a group velocity of 1.3 km s^−1^ can be seen in the positive and negative lag times of the CCFs. These signals propagate southwards (positive lag time) and northwards (negative lag time), with comparably large amplitudes. However, in the southern part of the line, two signals, with larger amplitudes than the noise level, propagate with group velocities of 1.5 and 0.7 km s^−1^ (blue and red triangles, respectively, in Fig. 2a). Here, we refer to regions at water depths of ~2 km in the northern part and ~4 km in the southern part as WD2 and WD4, respectively. Similar features can be seen at WD4 of Lines C and D (Supplementary Fig. 1b and Fig. 2b), while the northwards propagation at WD2 of the lines are stronger than the southwards propagation.

The CCFs for longer separation distances at WD2 show that wave propagations resemble the cases for the shorter separation distance, such as for the strong signals propagating northwards along Lines C and D (Fig. 3e,f), and northwards and southwards along Line A (Fig. 3c). The group velocity of the signal is 1.3 km s^−1^. At WD4, the strong southwards propagation can only be seen along Lines A, B and C, indicating weak northwards propagation for a long distance. The group velocities are within a range of 0.7–1.5 km s^−1^ (Fig. 3g–i). The strong directivity of wave propagation seems to reflect the seismicity around the Nankai Trough. The eastern seismicity (pink dots^23^ within box [S] in Fig. 3a) generates waves propagating northwards at WD2 and southwards at WD4 along Lines C and D, whereas the western seismicity (pale blue dots^24^ within boxes [N] and [S] in Fig. 3a) induces waves propagating northwards and southwards at WD2 and southwards at WD4 along the Line A. These features are summarized in Fig. 3k. The observed group velocities and those based on the normal mode calculations for appropriate one-dimensional structural models imply that the observed signals are persistently and randomly excited ACR waves (Methods; Supplementary Figs 2–4).

### Numerical simulations for the excitation of ACR waves

To explore seismic velocity models and excitation source models that can reproduce the above observations, we carried out 2D numerical simulations on the basis of a finite difference method with rotated staggered grids (RSGs)^25^. We consider three cases for excitation mechanisms that may generate persistent ACR waves: (1) the Rayleigh wave incidence from land, (2) generation of ocean-acoustic waves at the sea surface and their conversion to seismic waves at the seafloor by a mechanism similar to the mechanism generating microseisms (Longuet-Higgins^7^) and (3) seafloor disturbance resulting from randomly and persistently excited nearby submarine earthquakes. In case (1), ACR waves are generated by the conversion of continental Rayleigh waves upon their incidence to the oceanic region. In all the three cases, the sources are simply modelled by random vertical single forces^26^. In case (3), we applied random vertical forces inside the box in Fig. 4a, which simulates the seismic activity around the Nankai Trough^23^,^24^ (Methods).

After extensive numerical simulations, we found that the CCFs for case (3) best describe our observations. In case (1), the CCFs show a monochromatic wave with a velocity of ~1.1 km s^−1^ that propagates only southwards throughout the model space (Supplementary Fig. 5b,c), which is identified as a Scholte wave^27^ with its energy at the seafloor (Supplementary Fig. 5a). However, the observed CCFs do not show the characteristics of this wave.

In case (2) (Supplementary Fig. 5e), the CCFs show several groups of signals with a common apparent velocity of ~4 km s^−1^, which are interpreted as multiple reflections of P waves between the sea surface and seafloor. This phase can be identified in the observed CCFs with small amplitudes (arrows in Supplementary Fig. 1d), indicating that sources at the sea surface may slightly contribute to the production of ambient noise observed at the seafloor at WD2.

In case (3), the CCFs show both the ACR wave with a group velocity of 1.3 km s^−1^ and multiple reflections with an apparent velocity of ~4 km s^−1^ for WD2 (Fig. 4c), and the ACR waves with group velocities of 0.9–1.5 km s^−1^ for WD4 (Fig. 4d). Moreover, the synthetic CCFs for an inter-station distance of 5 km nicely simulate the northwards propagation at WD2 (red triangle at the top of Fig. 4b) and the southwards propagation at WD4 (red and blue triangles at the bottom of Fig. 4b). Thus, synthetic CCFs in this case are in good agreement with the observations in Line C (Supplementary Fig. 1b and Fig. 3e) and Line D (Figs 2b and 3f). Similarly, if we put random sources within the box in Supplementary Fig. 6a (Methods), the synthetic CCFs (red triangle at the top of Supplementary Fig. 6b) can simulate the observed signal propagating southwards at WD2 along Line A. Although the travel times are slightly different between the observed and synthetic signals, they can be adjusted by reducing model *V*s and the thickness of sediments at WD4. Seismic sources would thus be appropriate for the persistent generation of ACR waves in the Nankai Trough region.

## Discussion

Our results indicated that the observed features in the CCFs in the Nankai subduction zone would be mostly explained by ACR waves due to seismic sources. However, it is necessary to consider other mechanisms, including sources at the sea surface as suggested in case (2), to fully reproduce the features of the observed ACR waves. For instance, Fig. 3g–i show weak signals propagating northwards up to a distance of 40 km, which cannot be explained by seismic sources because of low seismic activity south of the array (Fig. 3). We suggest two possible mechanisms that persistently excite the northwards propagation at WD4. The first one is that excitation sources are located at the sea surface because the simulation result in case (2) shows the northwards propagation at distances shorter than 10 km at WD4 (Supplementary Fig. 5f), although it could not completely reproduce the observed feature. The second one is that the earthquake-excited ACR waves propagating southwards are scattered or/and reflected by complicated bathymetry and sediment structure south of the array^28^,^29^,^30^ (for example, at a region in the latitude range of 31.2°N–32.0°N in Fig. 3), and then the converted waves propagate northwards. To evaluate the efficiency of these two mechanisms, a full wavefield modelling taking into account scattering and reflection relevant to a realistic seismic structure would be required.

A normal mode search for phase velocities in a range of 0.6–3.5 km s^−1^ resulted in the first nine Rayleigh modes at 1 Hz and further higher modes at higher frequencies (Supplementary Fig. 4i,j). Which modes are efficiently excited depends on source mechanisms and locations. Although it would be difficult to identify which modes are dominant in the ACR waves obtained in this study, some constraint may be given from the calculated phase and group velocities of the normal modes. The first four Rayleigh modes, including the fundamental mode, have phase velocities close to 1.5 km s^−1^ in some frequency range, and such modes do not easily attenuate due to the high seismic Q of the water layer, and propagate long distances with little attenuation^31^,^32^. Therefore, we searched for the modes at 0.7–2.0 Hz with phase velocities close to 1.5 km s^−1^, within a range of 1.45–1.55 km s^−1^, and with group velocities consistent with the observed group velocities. The calculated group velocities are 1.3–1.4 km s^−1^ for WD2 and 1.3–1.5 km s^−1^ for WD4 in the case of a sediment *V*s of 1.32 km s^−1^ (Supplementary Fig. 4k–n), while they are 0.7–1.3 km s^−1^ for WD2 and 0.7–1.5 km s^−1^ for WD4 in the case of a sediment *V*s of 0.92 km s^−1^ (Supplementary Fig. 4o–r). Comparing these results with the observations, it seems that *V*s of the sediments is close to 1.32 km s^−1^ for WD2 (Supplementary Fig. 4l) and 0.92 km s^−1^ for WD4 (Supplementary Fig. 4r). Moreover, the calculated Rayleigh modes at 0.7–2.0 Hz correspond to the second-to-fifth higher modes in the case of a sediment *V*s of 0.92 km s^−1^ for WD4 (Supplementary Fig. 4q), and the second-to-fourth higher modes in the case of a sediment *V*s of 1.32 km s^−1^ for WD2 (Supplementary Fig. 4k). Considering the fact that the four gravest modes with phase velocities near 1.5 km s^−1^ propagate long distances^31^,^32^, the ACR waves observed in this study presumably consist of some of the first several higher modes that have the phase velocities at this frequency range.

Another important factor is the time required for stacking the CCFs to retrieve the ACR wave. This is related to the persistence of the excited wave. The seafloor ambient noise is composed of a number of coda waves associated with high seismic activities around the Nankai Trough (Supplementary Fig. 7a,b), and the coda waves in the relevant frequency range seem to consist mainly of non-attenuated ACR waves. Correlating ambient noises observed in this region, the daily stack is sufficient for retrieving the ACR waves, but retrieval from the hourly stack is unstable (Supplementary Fig. 7c,d). This indicates that the earthquake-excited ACR wave possibly dominates the short-period seafloor noise in this region and this phenomenon may also occur at other subduction zones where seismic activity is high^33^,^34^,^35^.

We found that the CCFs show the ACR waves at a frequency band of 0.7–2.0 Hz, and the amplitude pattern is in good agreement with the seismicity in the Nankai subduction zone. The comprehensive feature of the observations, especially in amplitude directivity, was well described by the earthquake-excited ACR waves, and the remaning could be explained by excitation sources located at the sea surface and scattering/reflection effects of the earthquake-excited ACR waves. Although excitation sources of background noises reported in previous studies have been mostly related to fluid motions, we found in this study that earthquakes occurring inside the solid Earth could be responsible for the persistent excitation in a seismically active region like the Nankai subduction zone.

## Methods

### Construction of CCFs

**Figure 1:**
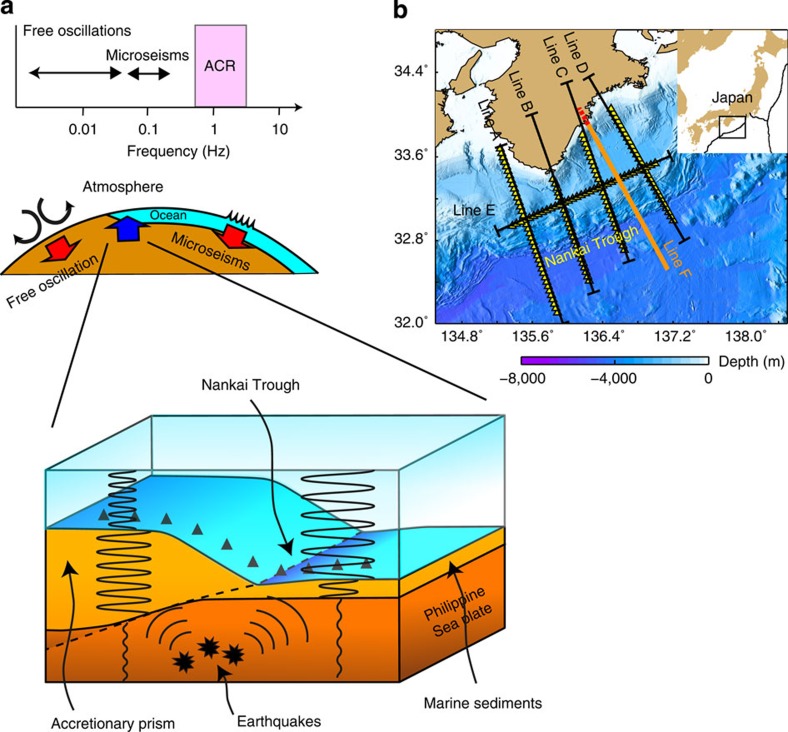
Schematic of longer-period incessant waves and ACR waves. (**a**) Sketch showing the characteristics of frequency and excitation source for persistent waves. The energy of the ACR wave is within the ocean and marine sediments, and the wave is excited by seismic signals. (**b**) Locations of the stations (yellow triangles), Lines A–E and Line F showing the profile of P-wave tomographic velocity model used in this study^20^.

For continuous hydrophone records, the absolute values of pressure greater than 0.03 Pa were reset to zero, to remove the effect of energetic signals, such as body waves from earthquakes. The CCFs were calculated using a time series length of 600 s. The time domain CCF was calculated through the inverse Fourier transform of the normalized cross spectrum, *C*_1,2_*(ω)*, in the frequency domain that is written as


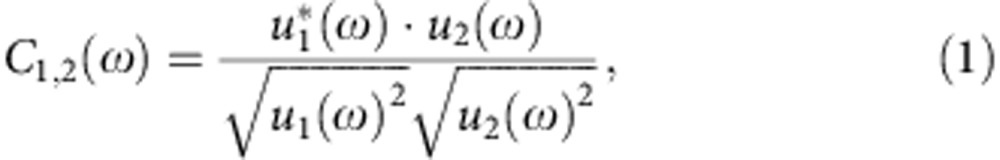


where *u*_1_*(ω)* and *u*_2_*(ω)* are continuous pressure records observed at receivers 1 and 2, and the asterisk (*) indicates a complex conjugate. We stacked the CCFs over an observation period of ~2 months. In this study, two approaches were adopted to align the resulting CCFs. In the first approach, we aligned only the CCFs using two adjacent stations, whose separation distance was 5 km along Lines A–E (Fig. 1b). This alignment of CCFs is useful for inquiring into the spatial variation of the wavefield at an equal incremental distance. In the second approach, we calculated CCFs for all combinations along each line: the stations indicated by orange and yellow triangles in Fig. 3a were used for the plots of WD2 (Fig. 3c–f) and WD4 (Fig. 3g–j), respectively; the trench normal profiles, Lines A–D, are divided into the shallower and deeper segments, WD2 and WD4, depending on the water depth. The CCFs, using two stations with the same separation distance, were then stacked. They were aligned as a function of separation distance. This alignment displays wave propagations as a function of time for long distances. When calculating CCFs in the NS-trending Lines A–D for both approaches, the record observed at the southern station of a station pair is reference one for use in equation (1). In the case of the EW-trending Line E, the record observed at the western station of a station pair is the reference. This indicates that waves that emerged in the positive lag time propagate northwards along Lines A–D and eastwards along Line E.

We summarized the notes on the CCFs obtained in this study below.

1. The numbers of CCFs were low at WD2 of Line B (Fig. 3d) and WD4 of Line D (Fig. 3j); therefore, we did not discuss the features of signals for these regions.
2. The resulting CCFs show that the signals propagating westwards and eastwards have comparable amplitudes not only at WD2 (Supplementary Fig. 1c), but also at WD4 (Supplementary Fig. 1e). This observation allows us to discuss amplitude variations of the signals propagating along the north–south direction, assuming uniformity in the east–west direction.
3. Several signals with velocities of ~4 km s^–1^ were extracted for WD2 (Supplementary Fig. 1d). This signal would correspond to multiple reflections of P waves between the sea surface and seafloor, with an incident angle of about 20°. The time interval of each signal, for example, positive peak-to-positive peak, was ~2.5–3.0 s. Assuming this time was the two-way travel time of a P wave with the incident angle, the water depth was estimated to be 1.7–2.1 km using a hydroacoustic wave speed of 1.5 km s^−1^. This estimation is in good agreement with the water depth at WD2. This phase can be reproduced by the numerical simulations in case (2) (Supplementary Fig. 5e), and case (3) (Fig. 4c). This implies that the multiple reflections observed in Supplementary Fig. 1d may be partly excited by microseisms (case 2).
4. Signals could not be observed in the CCFs using horizontal components, while the CCFs using vertical components show the features for the ACR wave propagations. This may indicate that ambient noise in the horizontal components at the seafloor is mainly dominated by shear wave resonances^36^,^37^.

### Response of hydrophone

The hydrophone used in this study can reliably record signals at frequencies higher than 2 Hz, although we used the frequencies of 0.7–2.0 Hz. It may be possible to check the detectability of seismic signals in this frequency range by comparison with the records of short-period seismometers (4.5 Hz) installed at the same place. As shown in Supplementary Fig. 8, the direct P wave and its water reverberation from a deep earthquake with a magnitude of 3.8 seemed to be observed well by hydrophones and short-period sensors at 0.7–2.0 Hz. We calculated CCFs using the direct P-wave portion observed at two hydrophones (red line in Supplementary Fig. 8d) and two seismometers (black line in Supplementary Fig. 8d). We used equation (1) for the CCF calculation with a time window of 6 s, which is delineated by a solid black line in Supplementary Fig. 8d. As a result, the peaks of 0.3 s consistently emerged in the CCFs from different instruments, that is, hydrophones and seismometers, which represent the differential travel time of a direct P wave between the two stations. This observation guaranteed the use of equation (1) with records of the hydrophone and the short-period sensors used in this study.

### Estimation of group velocities

**Figure 2:**
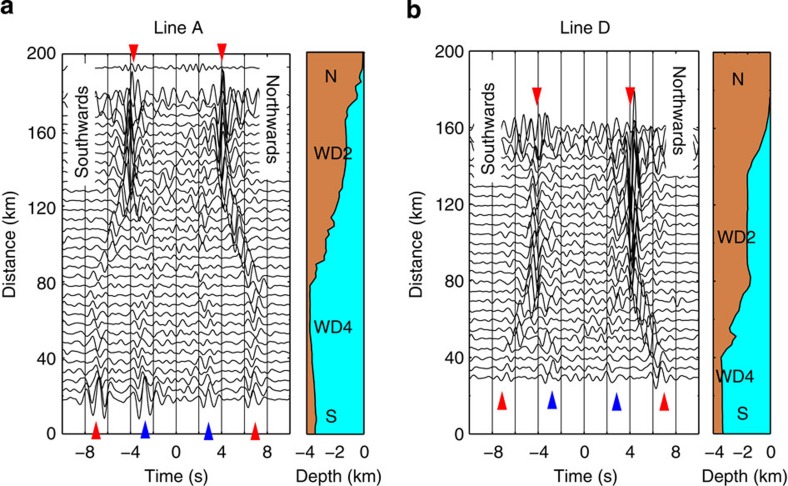
Observed CCFs for a separation distance of 5 km. (**a**) CCFs using records of two adjacent stations with bathymetry along Line A. Red and blue triangles indicate the extracted ACR waves. The signal that emerged in positive/negative lag time propagates northwards/southwards. (**b**) Same as **a**, except for Line D.

**Figure 3:**
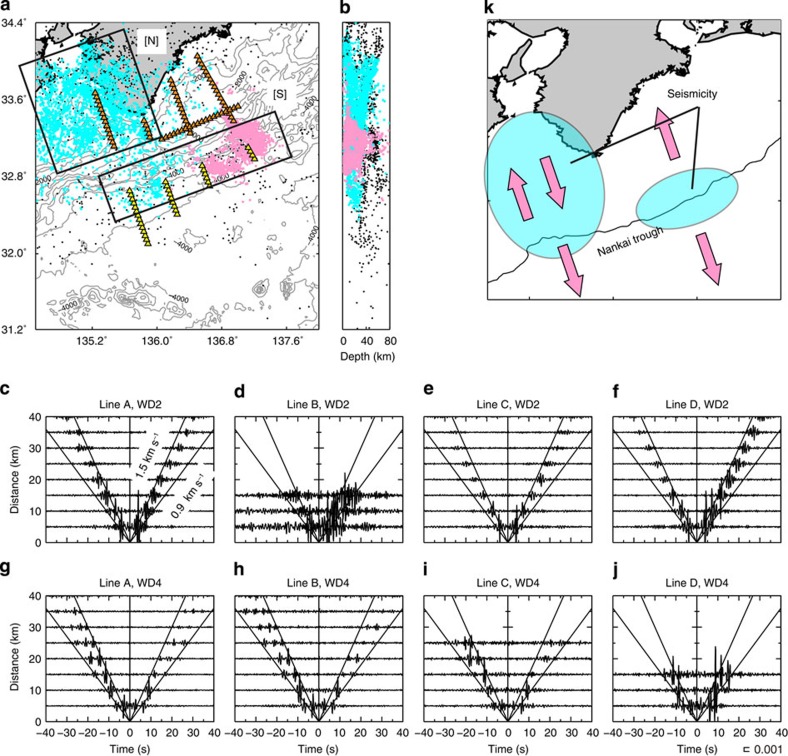
Seismicity and propagation direction of strong ACR waves. (**a**,**b**) Pale blue and pink dots represent the seismicities that occurred during the periods of December 2003–December 2007 (ref. 24) and January 2011–February 2013 (ref. 23), respectively. These two hypocentre data sets were determined by using ocean bottom records. Black dots correspond to the hypocentres occurred during January 2008–December 2013, determined by Japan Meteorological Agency (JMA). Boxes [N] and [S] roughly indicate regions for high seismic activity. (**c**) CCFs for all combinations of the stations indicated by orange triangles along Line A in **a**, which are aligned as a function of separation distance. Two solid lines in positive and negative lag times represent the reference velocities of 1.5 and 0.9 km s^−1^. Positive/negative lag time corresponds to northwards/southwards propagation. (**d**–**f**) Same as **c** except for Lines B, C and D at WD2. (**g**–**j**) Same as **c**–**f** except for the CCFs using yellow triangles at WD4. (**k**) The interpretation of (**a**–**j**). Pale blue region corresponds to the seismicity. Pink arrows represent the propagation directions of the strong ACR waves.

We estimated group velocities using CCFs at WD2 and WD4, with a frequency–time analysis^38^ in the frequency range of 0.5–2.5 Hz. As a result, in the frequency range of 0.7–2.0 Hz, the group velocity for WD2 was estimated to be 1.3 km s^−1^, while the velocity for WD4 expands to 0.7–1.5 km s^−1^, in which the signal with a group velocity of 0.9 km s^−1^ is strongest. These estimates are consistent with wave propagations displayed, for example, in Figs 2, 3, and in Supplementary Fig. 1.

### Velocity models for numerical simulations and DISPER80

The velocity model used in the simulation was created by referring to a P-wave tomographic velocity model along Line F^20^ (Fig. 1b). The acoustic velocity in the seawater was calculated by using a sound speed profile^39^, although our numerical simulation is not affected by the gradient of the velocity profile because the wavelength used is comparable to the water depth. *V*p is replaced by 3.3 km s^−1^ and *V*p*/V*s is set to 2.5, when *V*p is slower than 3.3 km s^−1^ below the seafloor. This means that *V*s immediately below the seafloor is constant at 1.32 km s^−1^. At greater depths, at which *V*p is larger than 3.3 km s^−1^ in the velocity model, *V*p*/V*s is set to 1.73 without changing *V*p from the original velocity model. The density is calculated by using the value of *V*p and a previously developed empirical relation^40^, which is applicable over the range 1.5<*V*p<8.5. The resulting velocity models for *V*p and *V*s are displayed (Supplementary Fig. 3a,b), and the profiles for *V*p, *V*s, *V*p*/V*s and density at horizontal distances of 40 and 130 km (Supplementary Fig. 3a,b) are shown in Supplementary Fig. 3c–f. These two profiles, which have water depths of 2 km (WD2) and 4 km (WD4), are used for normal mode calculation through the DISPER80 (ref. 41). Although the size of the original velocity model is 176 km (distance) × 30 km (depth), we expanded the model space further to the NNW (north-north-west) direction, resulting in a total distance of 196 km. The added segment has the same parameters at the northern edge (Fig. 3a). The calculation is limited to 10 km in depth in the numerical simulation with finite difference. Moreover, to remove the effect of seafloor topography with a large dip angle in numerical simulations, we applied a 20 km moving average to the bathymetry.

### Normal mode calculation with DISPER80

Using an open normal mode calculation code, DISPER80 (ref. 41), we estimated the eigenfunctions of stress (*τ*_zz_) with respect to the two velocity profiles that correspond to WD2 and WD4 (Supplementary Fig. 3c–f). The searching range of phase velocity is between 0.6 and 3.5 km s^−1^ with an increment of 0.001 km s^−1^, and the range of frequency is between 10^−0.4^ and 10^0.6^ Hz (0.398–3.981 Hz) with an increment of 0.01 in a power of 10. When the solution satisfying the boundary conditions for the Rayleigh modes was obtained, we plotted the phase and group velocities in Supplementary Fig. 4a–d. As a result, the group velocity of the Rayleigh mode is concentrated around 1.3 km s^−1^ and slightly scattered to higher velocities for WD2, while the group velocity of the Rayleigh mode expands between 1.0 and 1.5 km s^−1^ for WD4. Changing *V*s from 1.32 to 0.92 km s^−1^, the group velocities of the concentrated modes became slower at WD2, and only the lower boundary of the group velocity was shifted to slower at WD4 (Supplementary Fig. 4e–h). Comparing the normal mode calculation with the observation result (Supplementary Fig. 2), it seems that *V*s of the sediments is close to 1.32 km s^−1^ at WD2 and 0.92 km s^−1^ at WD4, respectively, although we did not take into account the lateral variation of *V*s within the sediment layer in the numerical simulation.

In addition, the eigenfunction curves show that, except for the Scholte wave, the Rayleigh modes have stress (*τ*_zz_) within the ocean and marine sediments at 1 Hz (Supplementary Fig. 4i,j), with various group velocities that are mainly constrained by *V*s of sediments. Judging from this result and the fact that the group velocity of the observed signal that emerged in the CCFs varies with water depth, the *V*s and the thickness of sediments, we considered the observed signal in the CCFs as the ACR wave.

### Numerical simulations with a finite difference method

**Figure 4:**
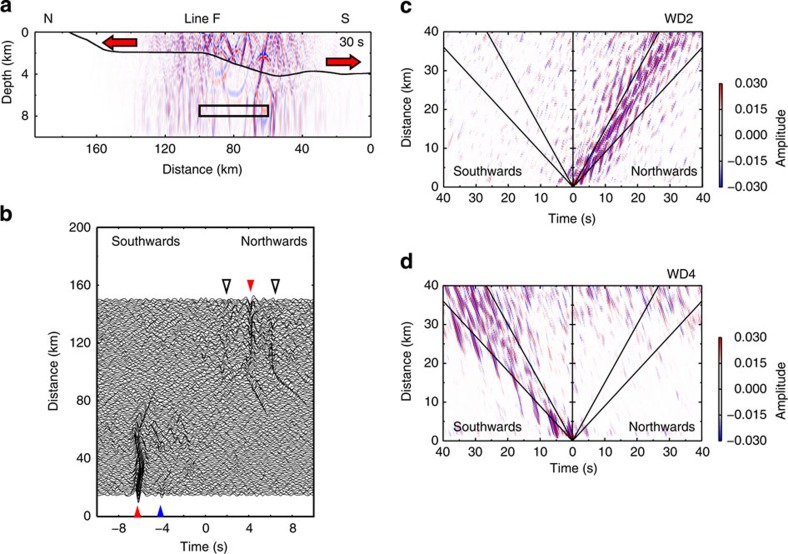
Synthetic CCFs for sub-seafloor sources. (**a**) Snapshot of vertical displacement wavefield associated with sub-seafloor sources applied within the box at the lapse time of *t=*30 s. Positive and negative amplitudes are displayed by red and blue colours. Red arrows represent the propagation directions of the ACR waves associated with the sources. (**b**) The synthetic CCFs using two stations with a separation distance of 5 km. Red and blue triangles represent signals that are consistent with our observation, and non-filled triangles indicate signals not observed (Methods). (**c**) The synthetic CCFs aligned as a function of separation distance of two stations for WD2 (100–150 km in horizontal distance in **a**). The two lines represent the reference velocities of 1.5 and 0.9 km s^−1^. (**d**) Same as **c** except for WD4 (10–60 km in horizontal distance in **a**).

We used a 2D finite difference method with a RSG^25^ for second order in time and space, which is capable of calculating the wavefield for the media including cracks and free surfaces. The calculation is performed in the displacement–stress scheme. Stations are set at the seafloor within a range of 10–150 km in distance with an interval of 0.5 km. The grid size is 10 m × 10 m. An absorbing boundary condition^42^ was used. We applied random vertical single forces varying in time as a Ricker wavelet with a central frequency of 2.0 Hz (with a maximum frequency of 2.9 Hz), within a box in Supplementary Fig. 5a (case 1), at the sea surface in a range indicated by the solid line in Supplementary Fig. 5d (case 2), and within a box in Fig. 4a (case 3). We applied vertical forces to the sea surface as excitation sources of microseisms^26^. We examined 300 shots in 120 s in case (2), and 30 shots in 120 s in cases (1) and (3). The time length for the numerical simulation is 160 s. Using synthetic stress records (*τ*_zz_) with a time length of 160 s, we calculated CCFs using two stations with a separation distance of 5 km, and CCFs for all combinations of stations within ranges of 10–60 km (for WD2) and 100–150 km (for WD4) in horizontal distance shown in Supplementary Fig. 3a,b. A bandpass filter of 0.7–2.0 Hz was applied to each CCF.

The minimum velocity of 1.32 km s^−1^ in our numerical simulations produces a wavelength of 0.455 km with 2.9 Hz, resulting in 45 grid points per minimum wavelength with a grid size of 10 m. It has been reported that the RSG calculation for at least 30 grid points per minimum wavelength provides results with sufficient accuracy for the cases of free surface and crack^43^,^44^. In addition, since we removed the effect of bathymetry with a large dip angle, by applying the moving average to the seafloor depth, the obtained wavefield was reliable.

In the resulting synthetic CCFs, some weak signals that did not emerge in the observed CCFs were generated in the numerical simulations (non-filled triangles in Fig. 4b and Supplementary Fig. 6b). Such a discrepancy may be because we adopted simple source- and seismic-velocity models. Mechanism solutions for earthquakes and lateral variations in *V*s may be required for reproducing the observations more properly.

### Location of earthquakes for the excited waves

Excited waves are slightly changed by the location of earthquakes. We applied random sources within the boxes in Fig. 4a and Supplementary Fig. 6a, referring to the seismicity indicated by boxes [N] and [S] in Fig. 3a. Before the determination of this location, we examined four earthquake locations shown in Supplementary Fig. 9a: (A) under the dipping seafloor near the coastline, (B) under the flattened seafloor at WD2, (C) under the dipping seafloor on the right north of the Nankai Trough and (D) under the flattened seafloor at WD4. Of the four cases, locations (A) and (C) could support our observations (Supplementary Fig. 9b,d), while locations (B) and (D) describe parts of the observed features (Supplementary Fig. 9c,e).

In our model, since signals impinging under dipping interfaces effectively generated ACR waves, we selected the regions around locations (A) and (C) for earthquake locations. For a more sophisticated simulation, the focal mechanism solution of earthquakes and the depth of earthquakes should be taken into account; this consideration would cause slowness of incident waves to the seafloor and variation of their wave types, such as P and S waves.

In addition, we used an incident plane wave, corresponding to teleseismic P waves, with a slowness of 0.08 s km^−1^. As a result, multiple reflections of P wave between the sea surface and seafloor are dominant in the resulting CCFs, implying that near-field earthquakes are necessary for the generation of ACR waves.

## Author contributions

T.To. performed data processing and numerical simulations, and prepared the manuscript with feedback and contributions from all the co-authors. Both T.To. and Y.F. contributed to the interpretations. T.Ta., K.O. and S.K. designed the seismic surveys and participated in the data acquisition. T.Ta. also participated in the data processing. Y.K. operated the project that conducted the seafloor observation in the Nankai subduction zone.

## Additional information

**How to cite this article**: Tonegawa, T. *et al.* Ambient seafloor noise excited by earthquakes in the Nankai subduction zone. *Nat. Commun.* 6:6132 doi: 10.1038/ncomms7132 (2015).

## Supplementary Material

Supplementary Figures Supplementary Figures 1-9
