# Supplementary material for: Ambient seafloor noise excited by earthquakes in the Nankai subduction zone
Source: Nat Commun. 2015 Jan 30;6:6132. doi: 10.1038/ncomms7132 (PMC4317493; doi:10.1038/ncomms7132)
Supplement: Supplementary Figures — 1-9 [file ncomms7132-s1.pdf]

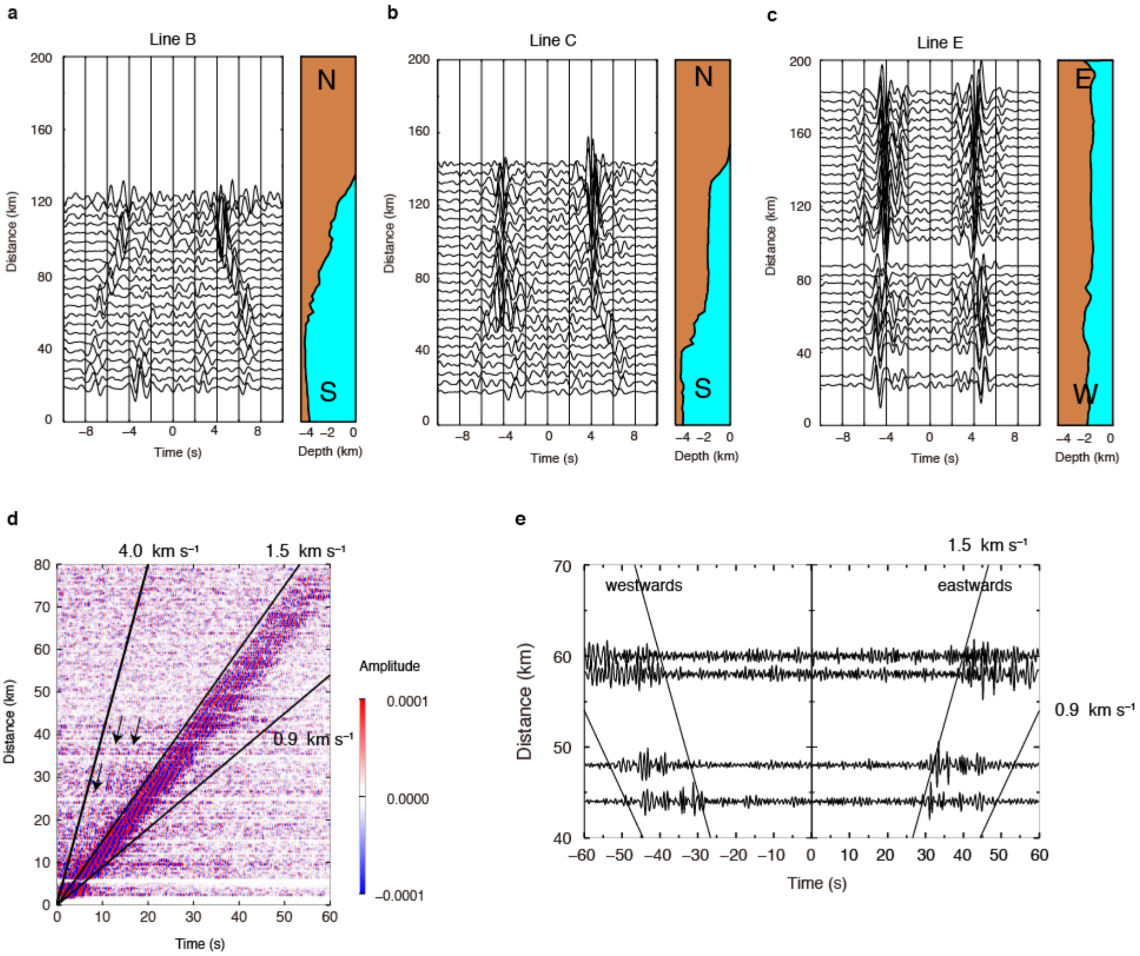

Supplementary Figure 1 | Observed CCFs for other lines and regions. (**a**–**c**) Same as Fig. 2a, except for Lines C–E. (**d**) CCFs for all combinations of stations indicated by orange triangles in Fig. 3a, which are aligned as a function of separation distance. Three lines represent the reference velocities of 4.0, 1.5, and 0.9 km s^{-1}. Arrows indicate multiple reflections of *P* wave (Methods). (**e**) CCFs using the stations indicated by yellow triangles in Fig. 3a, but the plotting CCFs were selected under a condition in which the azimuth from one station to another is limited to N65°E–N75°E. This is based on the direction of Line E, ~N70°E. Two lines represent the reference velocities of 1.5 km s^{-1}, and 0.9 km s^{-1}.

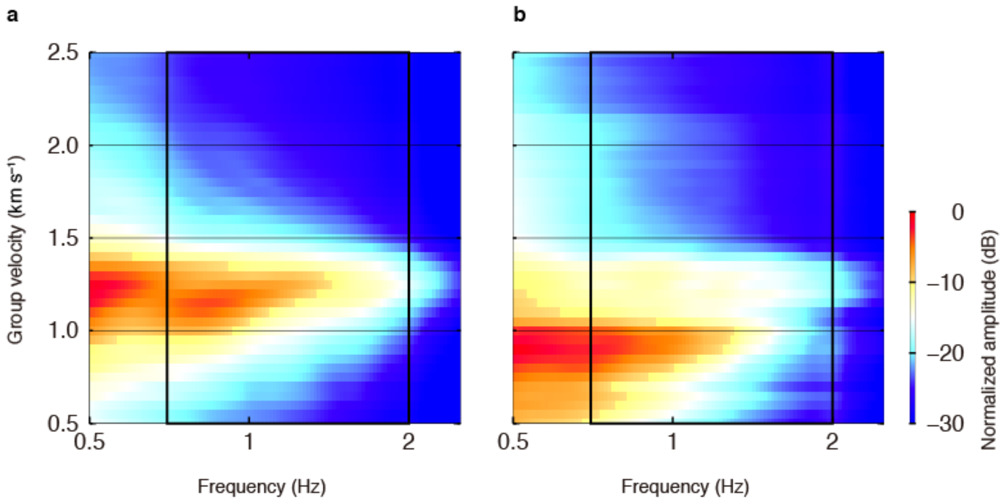

Supplementary Figure 2 | Estimation of group velocity for WD2 and WD4. (**a**) The frequency-dependent group velocity for WD2 estimated by the CCFs using stations indicated by orange triangles in Fig. 3a. The box represents the frequency range used in this study. (**b**) Same as Supplementary Fig. 2a, except for WD4.

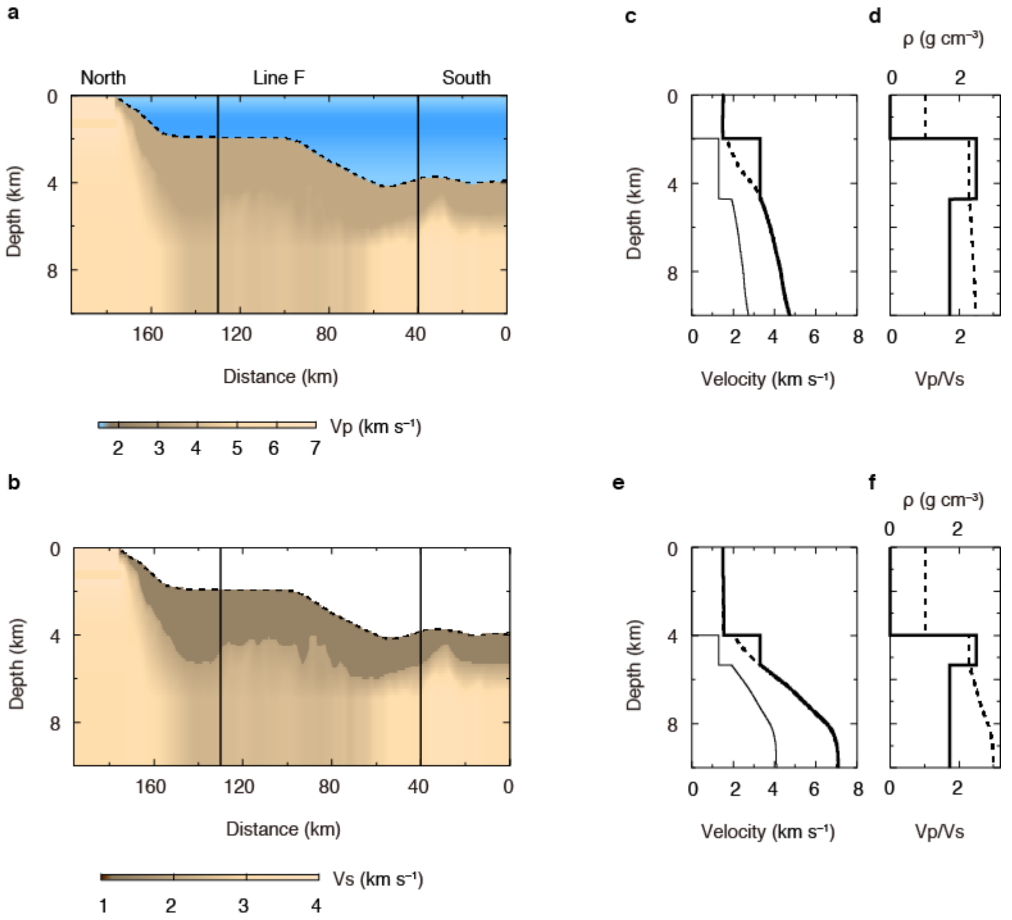

Supplementary Figure 3 | Velocity model in numerical simulations and DISPER80. (**a**) *Vp* model used in the numerical simulation, created by referring to a P wave tomographic model[20]. (**b**) *Vs* model used in the numerical simulation. (**c**) Solid, dashed, and thin lines represent *Vp*, the referred *Vp*[20], and *Vs* profiles at the horizontal distance of 130 km in Supplementary Figs. 3a and b (WD2). (**d**) Solid and dashed lines represent *Vp/Vs* and density profiles. (**e, f**) Same as Supplementary Figs. 3c and d, except for a horizontal distance of 40 km in Supplementary Figs. 3a and b (WD4).

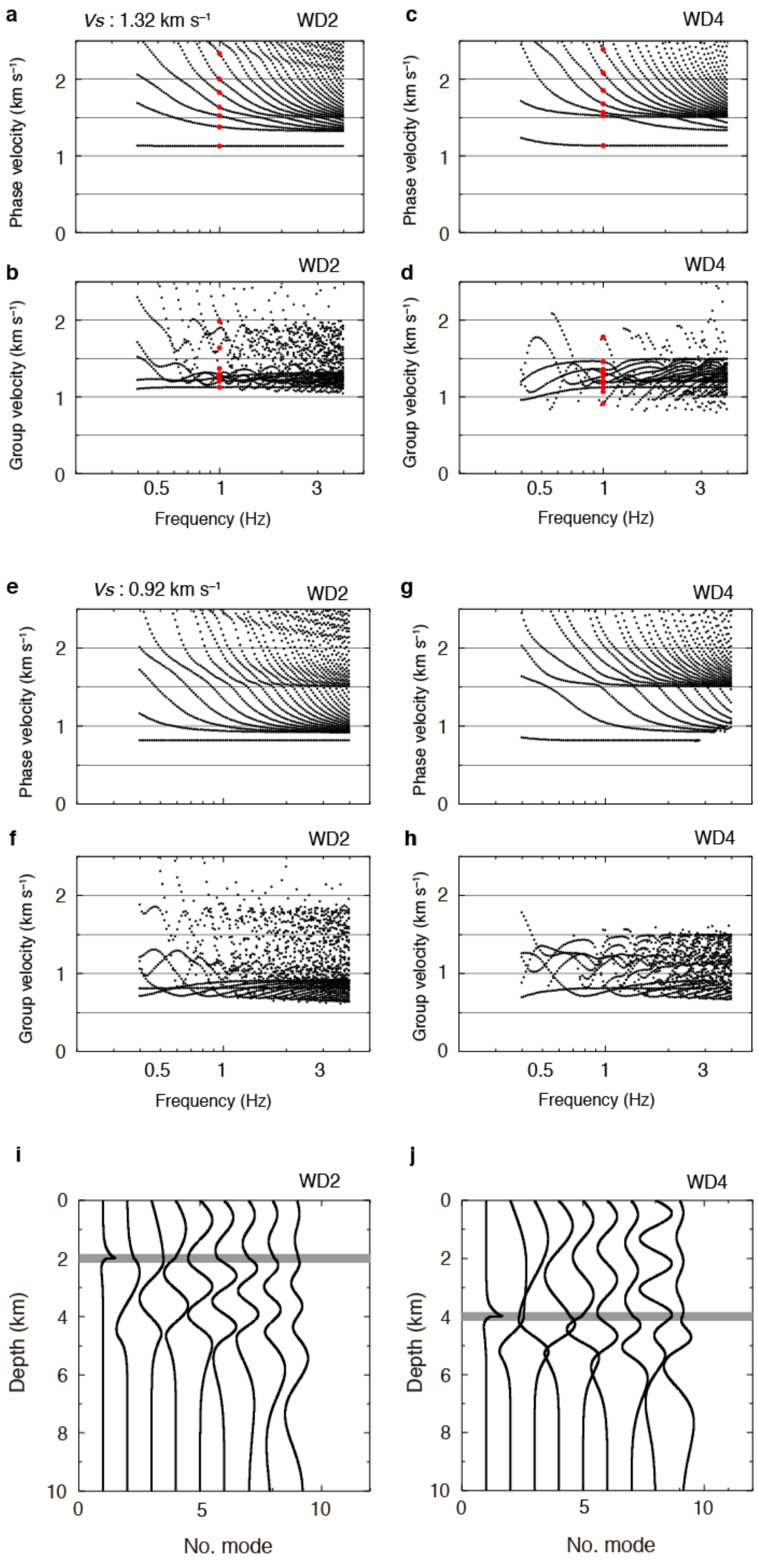

a
Vs : 1.32 km s⁻¹
WD2
Phase velocity (km s⁻¹)
c
WD4
Phase velocity (km s⁻¹)
b
WD2
Group velocity (km s⁻¹)
Frequency (Hz)
d
WD4
Group velocity (km s⁻¹)
Frequency (Hz)
e
Vs : 0.92 km s⁻¹
WD2
Phase velocity (km s⁻¹)
g
WD4
Phase velocity (km s⁻¹)
f
WD2
Group velocity (km s⁻¹)
Frequency (Hz)
h
WD4
Group velocity (km s⁻¹)
Frequency (Hz)
i
WD2
Depth (km)
No. mode
j
WD4
Depth (km)
No. mode

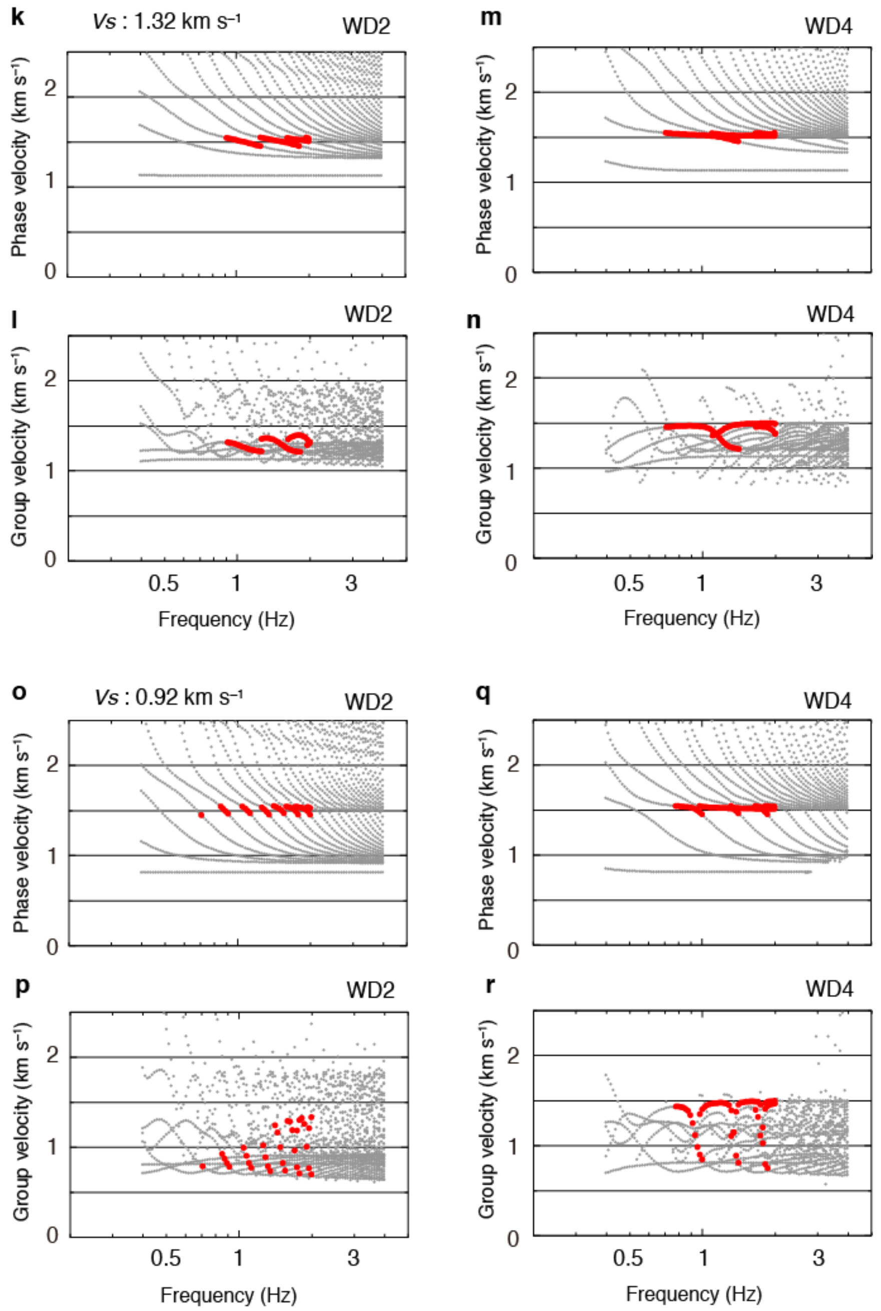

Supplementary Figure 4 | Phase, group velocities and eigenfunctions from DISPER80. (**a**, **b**) Phase and group velocities as a function of frequency for WD2, with *Vs* of 1.32 km s^{-1} in marine sediments. (**c**, **d**) Same as Supplementary Figs. 4a and b, except for WD4. (**e**–**h**) Same as Supplementary Figs. 4a–d, except for *Vs* of 0.92 km s^{-1} in marine sediments. (**i**, **j**) Eigenfunctions of stress (τ_{zz}) from fundamental to higher modes at 1 Hz, with increasing the number of mode. The obtained phase and group velocities are indicated by red dots in Supplementary Figs. 4a and b for WD2, and 4c

and d for WD4, respectively. (**k–r**) Same as Supplementary Figs. 4a–h, but red dots represent group velocities (**l**, **n**, **p**, **r**) of the Rayleigh modes with phase velocities of 1.45–1.55 km s^{-1} (**k**, **m**, **o**, **q**). Grey dots are the same as those in Supplementary Figs. 4a–h.

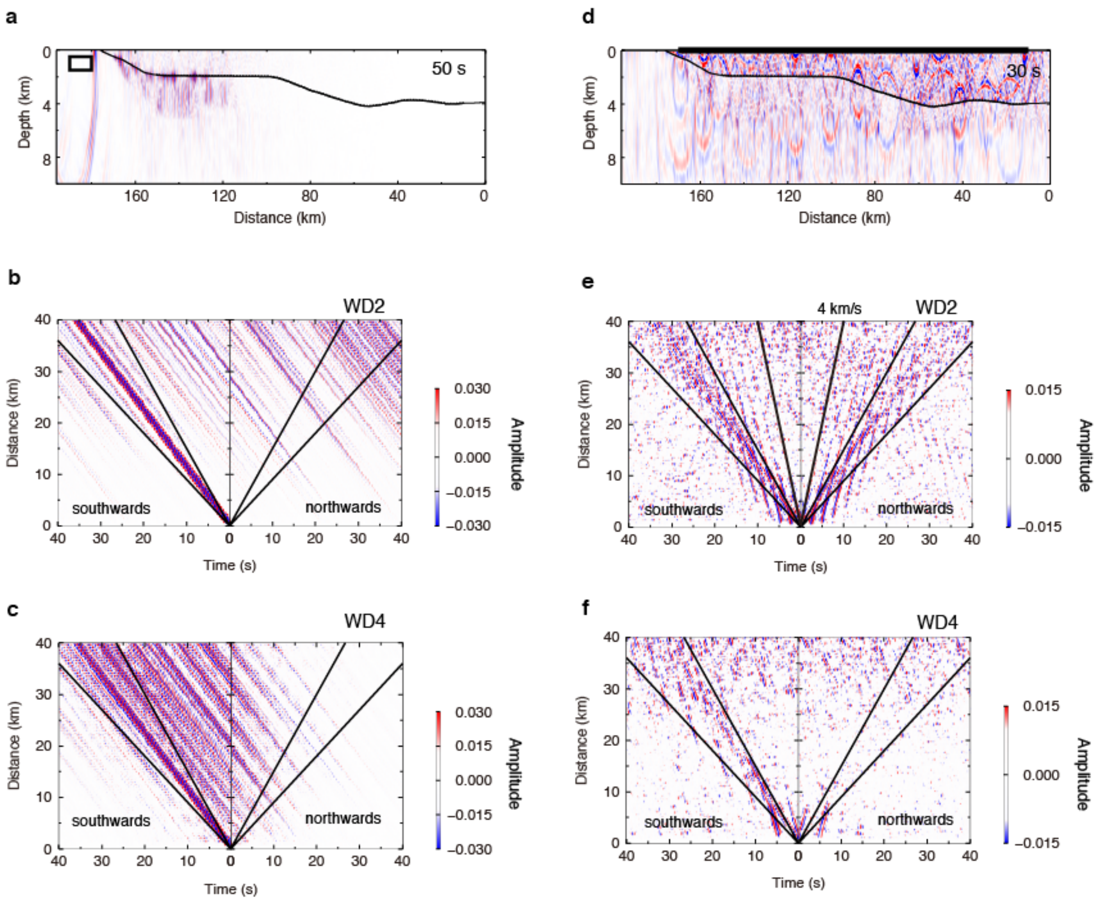

Supplementary Figure 5 | Synthetic CCFs for excitation sources related to microseisms. (**a**) Same as Fig. 4a, except for land sources applying within the box. The lapse time of the snapshot is t = 50 s. (**b**, **c**) Same as Figs. 4c and d, except for land sources with the reference velocities of 1.5 and 0.9 km s^{-1}. (**d**–**f**) Same as Supplementary Figs. 5a–c, except for sea surface sources applying along the solid line with the reference velocities of 4.0, 1.5, and 0.9 km s^{-1}. The lapse time of the snapshot is t = 30 s.

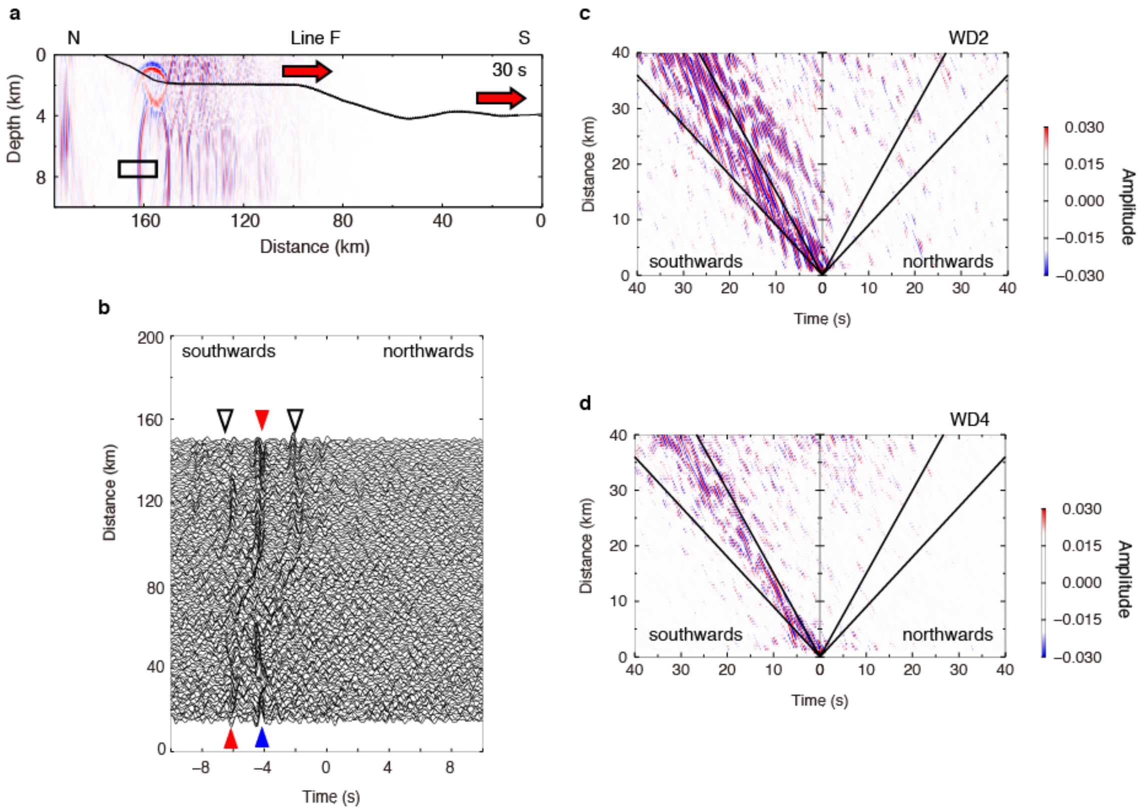

Supplementary Figure 6 | Synthetic CCFs for sources near the coastline. (a–d) Same as Fig. 4, except for sources applying within the box in Supplementary Fig. 6a.

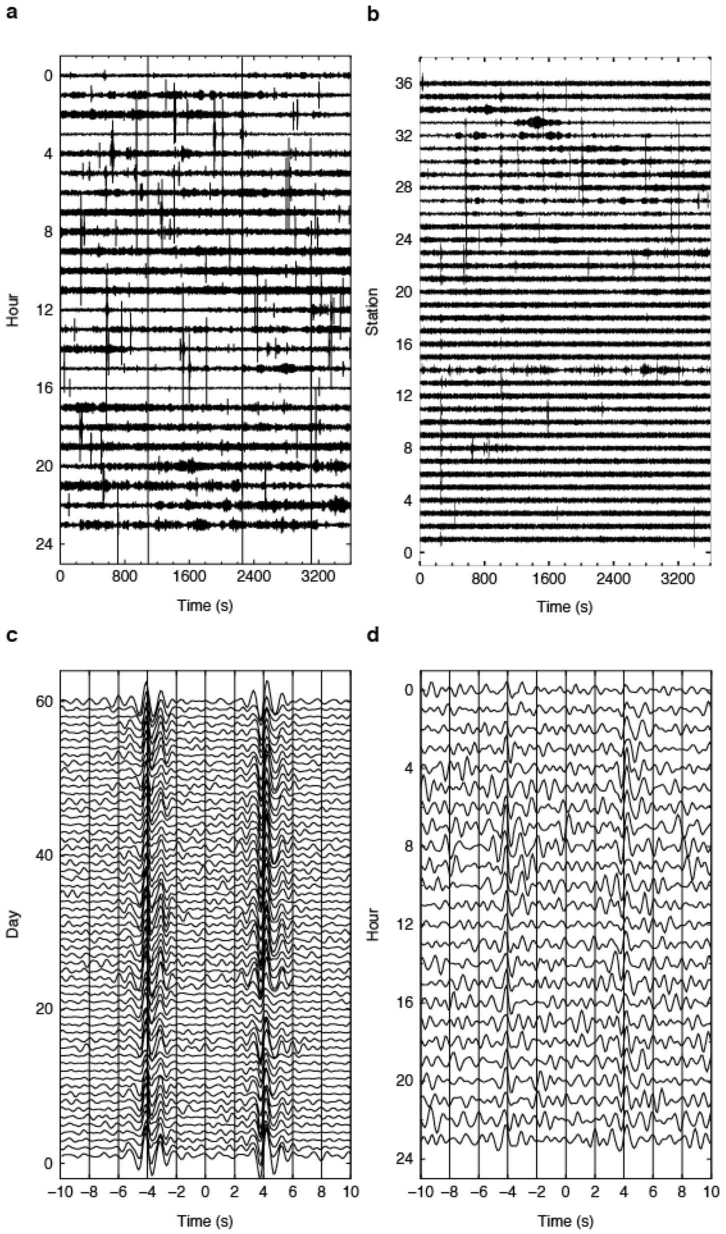

Supplementary Figure 7 | Extraction of ACR wave and seismic activity around Line A. (**a**) One-day seismogram with a bandpass filter of 1–8 Hz at a station S030, observed by the short-period sensor. (**b**) One-hour seismograms with a bandpass filter of 1–8 Hz at the stations along Line A (Fig. 1b), observed by short-period sensors. (**c**) Daily CCFs for two stations, S030 and S031. The locations of the two stations are shown in Supplementary Fig. 8a. (**d**) Hourly CCFs for S030 and S031.

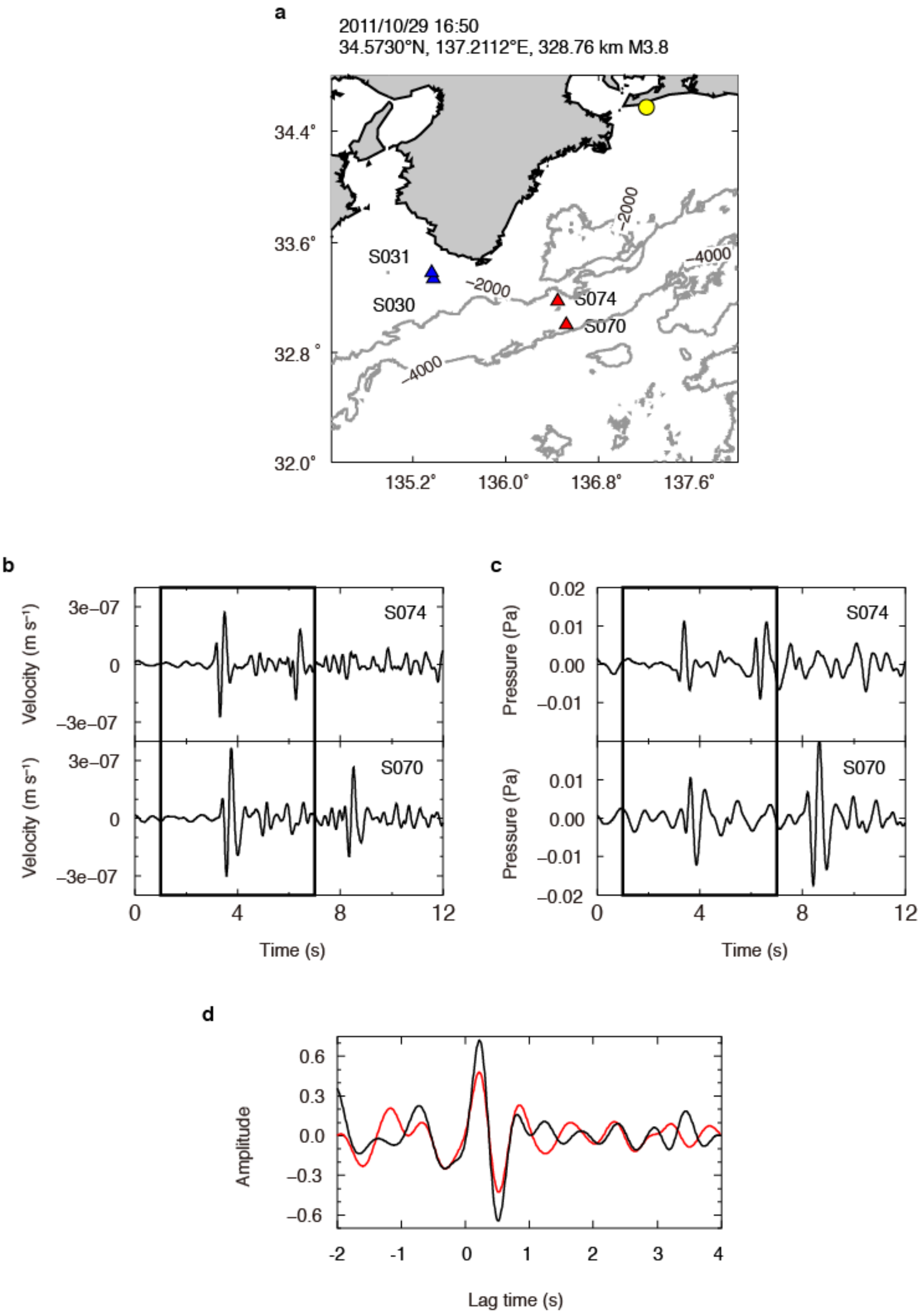

Supplementary Figure 8 | Comparison of CCFs obtained from hydrophone and short-period sensor. (**a**) Map showing locations of the stations, including S030 (blue triangle), S031 (blue triangle), S070 (red triangle), and S074 (red triangle), and the epicentre of a deep earthquake (yellow circle) that occurred on 29 October, 2011. (**b**) Seismograms of direct *P* wave of the deep earthquake at stations, S070 and S074, observed by a short-period sensor. The box indicates the time window for cross-correlation. (**c**) Same as Supplementary Fig. 8b, but for hydrophone records. (**d**) CCFs using two hydrophones (red line) and two short-period sensors (black line).

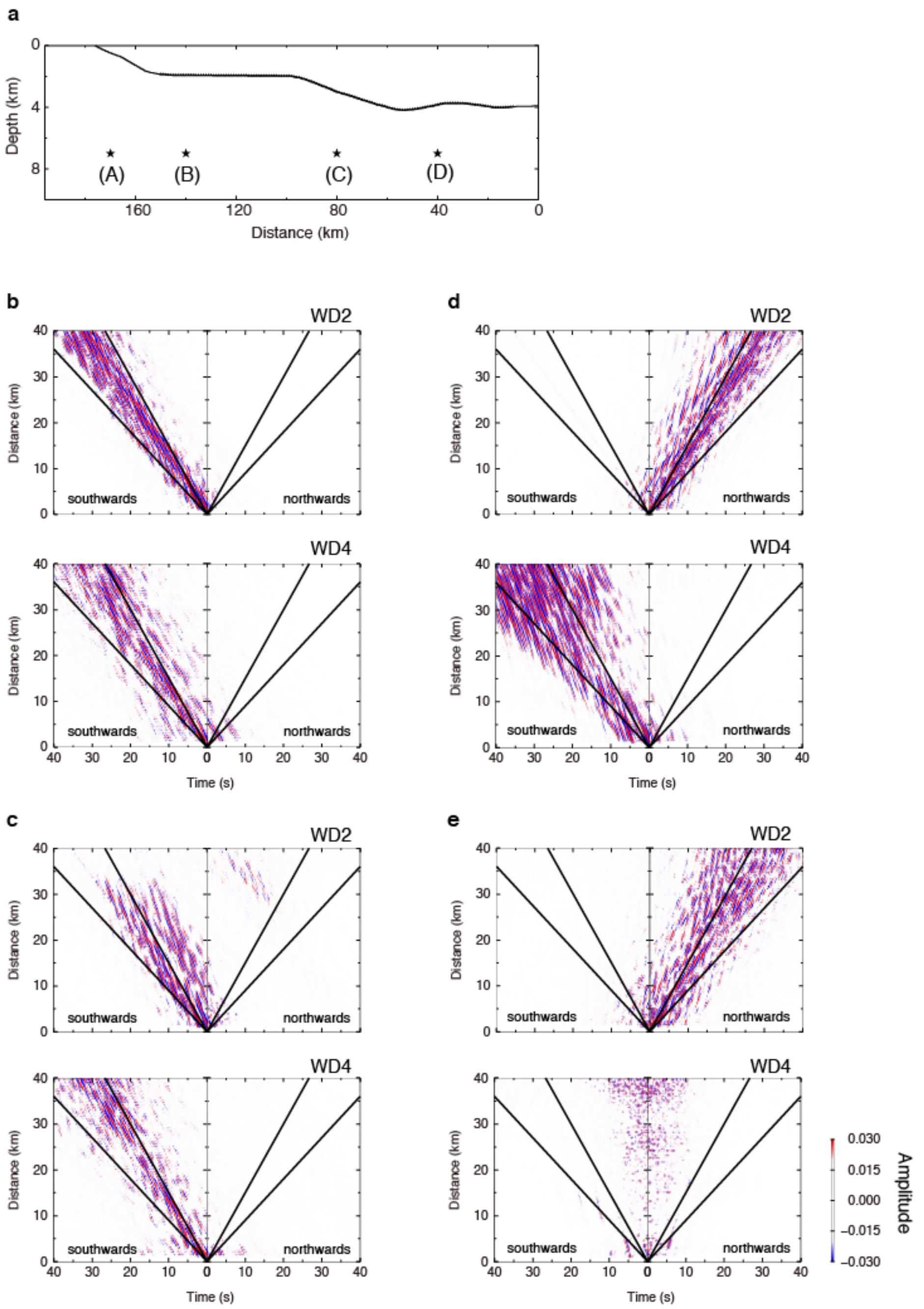

Supplementary Figure 9 | Variations of extracted waves according to source locations. (**a**) The locations applying vertical forces. (**b**–**e**) Same as Figs. 4c and d, except for locations (A), (B), (C), and (D).
